# Expression of Lymphatic Markers in the Berger’s Space and Bursa Premacularis

**DOI:** 10.3390/ijms22042086

**Published:** 2021-02-19

**Authors:** Seita Morishita, Takaki Sato, Shou Oosuka, Taeko Horie, Teruyo Kida, Hidehiro Oku, Kimitoshi Nakamura, Shinji Takai, Denan Jin, Tsunehiko Ikeda

**Affiliations:** 1 Department of Ophthalmology, Osaka Medical College, 2-7 Daigaku-machi, Takatsuki-City 569-8686, Osaka, Japan; infinity_s2000@yahoo.co.jp (S.M.); opt147@osaka-med.ac.jp (T.S.); s_osuka_0606@yahoo.co.jp (S.O.); opt168@osaka-med.ac.jp (T.H.); opt038@osaka-med.ac.jp (T.K.); opt025@osaka-med.ac.jp (H.O.); 2 Nakamura Eye Clinic, 3-6-22 Chuo, Matsumoto-City 390-0811, Nagano, Japan; nakamura-ganka01@tuba.ocn.ne.jp; 3 Department of Innovative Medicine, Graduate School of Medicine, Osaka Medical College, 2-7 Daigaku-machi, Takatsuki-City 569-8686, Osaka, Japan; pha010@osaka-med.ac.jp (S.T.); pha012@osaka-med.ac.jp (D.J.)

**Keywords:** bursa premacularis (BPM), Berger’s space (BS), podoplanin, lymphatic vessel endothelial hyaluronan receptor 1 (LYVE-1), fibrillin, lymph node, conduit system, initial lymphatics, anchoring filament

## Abstract

We previously reported that the bursa premacularis (BPM), a peculiar vitreous structure located above the macula, contains numerous cells expressing markers of lymphatic endothelial cells, such as podoplanin and LYVE-1. Herein, we examined the expression of lymphatic markers in the Berger’s space (BS), BPM, and vitreous core (VC). BS, BPM, and VC specimens were selectively collected in macular hole and epiretinal membrane patients during vitrectomy and were then immunostained with antibodies for podoplanin, LYVE-1, and fibrillin-1 and -2. By visualization using triamcinolone acetonide, the BS was recognized as a sac-like structure with a septum located behind the lens as well as BPM. Those tissues adhered to the lens or retina in a circular manner by means of a ligament-like structure. Immunostaining showed intense expression of podoplanin and LYVE-1 in the BS. Both BS and BPM stained strongly positive for fibrillin-1 and -2. The VC was faintly stained with antibodies for those lymph-node markers. Our findings indicate that both BS and BPM possibly belong to the lymphatic system, such as lymph nodes, draining excess fluid and waste products into lymphatic vessels in the dura mater of the optic nerve and the ciliary body, respectively, via intravitreal canals.

## 1. Introduction

The vitreous body is a hydrogel with very high water content ranging from 98 to 99%, which impedes the use of usual histological methods to determine the structure [1,2]. Thus, most of the fine structures of the vitreous remain obscured. In 1971, using a modified slit-lamp camera, Eisner observed membranous structures in the vitreous, named “tractae” (i.e., tracts), that coursed from the region around the lens in a circumferential pattern, parallel to the vitreous cortex, and inserted at the posterior pole [3,4]. Worst and Los subsequently reported visualizing approximately 120 cisterns within the vitreous body after injecting it with colored ink (Magic^®^ Color, Royal Sovereign; red, blue, and white) [5] and postulated that the tracts reported by Eisner constituted the wall of the cisterns, all of which interconnected in the middle of the vitreous, forming the cisternal system of Worst [6]. Both Eisner and Worst stored the eye specimens in a balanced salt solution at 4 °C for several days before initiating dissection of the eye, which facilitated removal of the retina from the vitreous [5].

In 1975, Worst first reported the bursa premacularis (BPM) to be a part of the cisternal system with a peculiar sac-like structure located in the premacular vitreous [5,7]. Worst conjectured that the BPM might protect the macula in both hydrodynamic and biochemical senses [8], although the anatomical structure and physiological role of the BPM have yet to be fully elucidated.

Our recent study exhibited that the vitrectomy specimens of the thin tissue remaining on the macula after induction of posterior vitreous detachment (PVD) contained numerous cells, including those expressing lymphatic markers, such as podoplanin and lymphatic vessel endothelial hyaluronan receptor 1 (LYVE-1) [9]. This thin tissue has generally been considered the remaining vitreous cortex. However, our findings indicated the possibility that it was the BPM and that it that might have properties similar to lymph nodes [9].

The Berger’s space (BS) is another accessory structure of the vitreous body, and it is a tiny anatomical gap located between the lens and the anterior hyaloid membrane (AHM) [10]. It has been reported that the BS is where various objects accumulate, such as blood, inflammatory cells, pigments, and proteinaceous materials including amyloid [11,12,13,14,15]. Using Vitreokontrast suspension (i.e., a dispersed suspension of nonorganic barium sulfate) with sequential removal of the anterior vitreous cortex up to the posterior lens capsule, Kislitsyna et al. showed the possibility that the retrolental bursa (sac) with an anterior wall attached to the lens existed, rather than the retrolental space (i.e., the BS), with no anterior wall (YouTube. Available at: http://www.evrs.eu/on-the-inner-side-of-lens-anatomic-and-topographic-features-of-anterior-vitreous-2/ (accessed on 31 May 2019)) [16,17].

In this present study, we examine the expression of lymphatic markers (podoplanin, LYVE-1, and fibrillin-1 and -2) in specimens of the BS (or its wall) and the BPM that were selectively collected during vitrectomy and report the results of immunostaining of these two tissues, with a discussion on the possibility that they might have functions similar to the lymphatic system, such as lymph nodes.

## 2. Results

### 2.1. Anatomical Similarities between the BS and the BPM Observed during Specimen Collection

**Figure 1:**
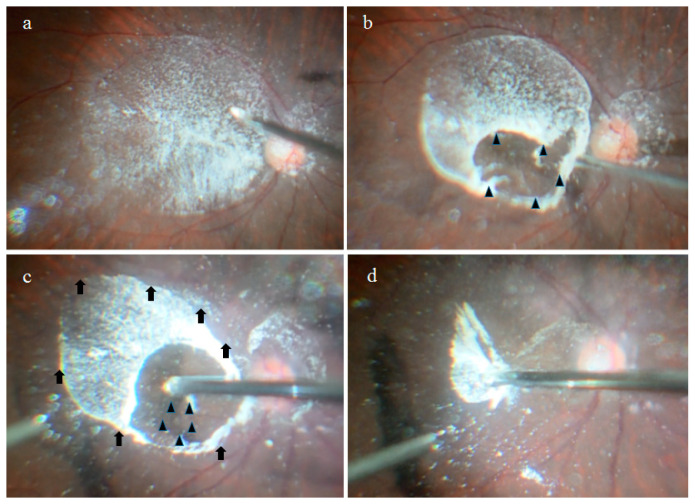
Surgical procedure used to obtain the bursa premacularis (BPM) specimens. Triamcinolone acetonide (TA) particles were firmly adhered to only the surfaces of the BPM and the Martegiani area. The BPM was observed to be an oval or round thin tissue with a size of 5 to 6 disc diameters on the macula (**a**). Two penetrating holes were easily created in the anterior and posterior walls of the BPM by surgically scraping the surface with a diamond scraper (**b**,**c**); black arrowheads). Firm adhesion with a circular ligamentous structure was observed in the periphery of the BPM (**c**; thin black arrows), and this adhesion extended to the Martegiani area that also firmly adhered to the retina around the optic disc. The combined specimen of the BPM and Martegiani area was then separated from the retina via aspiration of the edge of the hole in the anterior wall of the BPM with a vitreous cutter. The tissue specimen was then released into the vitreous and selectively collected with a vitreous cutter (**d**).

In all patients, the BPM was observed using triamcinolone acetonide (TA) to be an oval or round-shaped thin tissue with a size of 5 to 6 disc diameters on the macula (Figure 1a). Since the central part of the adhesion between the BPM and the retina was weak, holes were easily created in the anterior and posterior walls by surgically scraping with a diamond scraper (Figure 1b,c). The BPM was then removed from the retinal surface via aspiration of the edge of the hole in the anterior wall with a vitreous cutter. It was relatively easy to detach the central part of the BPM from the retina, even though strong adhesion with a circular ligamentous structure was observed in the periphery (Figure 1c; thin black arrows). This adhesion to the retina extended from the BPM to the Martegiani area with a septal structure. The Martegiani area was located around the optic disc, with a size of approximately 2 disc diameters. By further lifting the combined specimen of the BPM and the Martegiani area with a vitreous cutter, the circular adhesions of the specimen were removed. The specimen was then released into the vitreous cavity and was selectively collected by cutting into multiple small pieces with a vitreous cutter (Figure 1d). The time required for collection of the BPM specimen was approximately 1 min, with no increase in invasiveness or complications.

**Figure 2:**
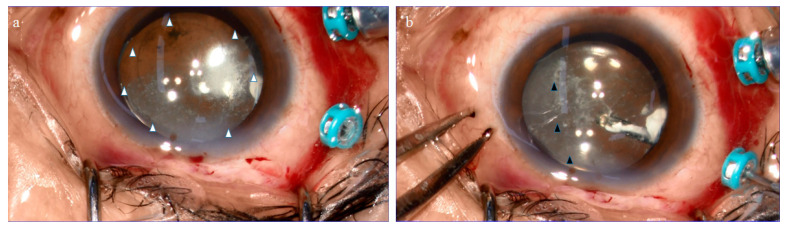
Surgical procedure used to obtain the Berger’s space (BS) specimens. TA was injected into the BS from behind during vitrectomy in order to visualize the configuration (**a**). The periphery of the BS was firmly adhered to the lens with a circular ligamentous structure, known as Wieger’s ligament (**a**; white arrowheads). A septum-like structure appeared to divide the BS into a two-thirds temporal and one-third nasal formation (**b**; black arrowheads). The BS specimen was then separated from the lens via aspiration of its posterior wall with a vitreous cutter. The specimen was then released into the vitreous cavity and selectively collected with the vitreous cutter.

The BS was visualized by injecting it with TA (Figure 2a). The injection of TA showed a septum-like structure dividing the BS into a two-thirds temporal and one-third nasal formation (Figure 2b; black arrowheads). The central part of the BS was easily detached from the lens capsule via aspiration with a vitreous cutter, even though a firm circular adhesion with a ligamentous structure was observed in the periphery. By further lifting the posterior wall of the BS with the vitreous cutter, the circular adhesion was removed. The BS specimen was then selectively collected after cutting it into multiple small pieces by use of the vitreous cutter. The intraoperative findings indicate that the BS and the BPM had similar elastic properties.

### 2.2. Immunostaining of the BPM and the Vitreous Core (VC) with Antibodies for Fibrillin-1 and -2

**Figure 3:**
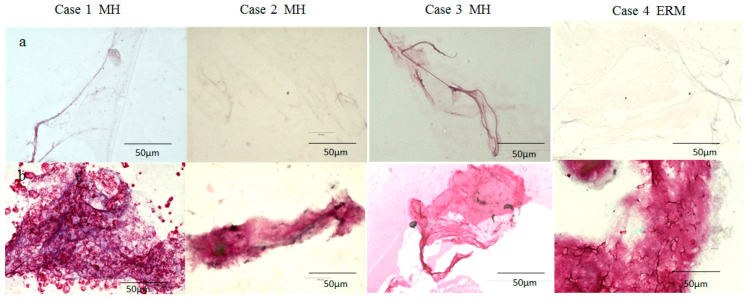
Immunostaining of the BPM specimen with antibodies for fibrillin-1. The vitreous core (VC) of patients with a macular hole (MH) and with an epiretinal membrane (ERM) was faintly stained with antibodies for fibrillin-1 (**a**), whereas the BPM was intensely stained (**b**).

**Figure 4:**
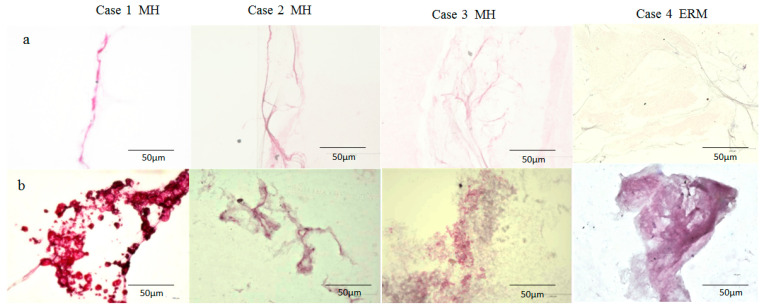
Immunostaining of the BPM with antibodies for fibrillin-2. Similar to fibrillin-1, the VC of patients with MH and ERM was faintly stained with antibodies for fibrillin-2 (**a**) whereas the BPM was intensely stained (**b**).

In Cases 1–4, the BPM specimen was intensely stained with antibodies for fibrillin-1 and -2, whereas the VC was faintly stained (Figure 3a and Figure 4a). While some BPM specimens were diffusely stained with antibodies for fibrillin-1 and -2 (Figure 3b: Cases 2 and 3; Figure 4b: Case 4), others exhibited an annular staining pattern (Figure 3b: Case 1; Figure 4b: Cases 1 and 3).

### 2.3. Immunostaining of the BS, BPM, and VC with Antibodies for Fibrillin-1 and -2, Podoplanin, and LYVE-1

**Figure 5:**
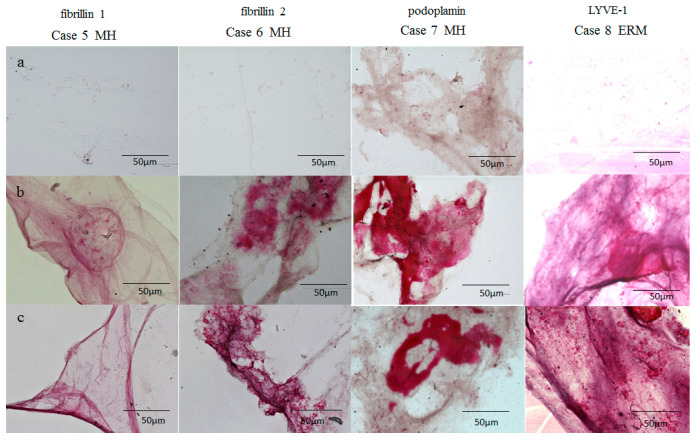
Immunostaining of the VC (**a**), BPM (**b**), and BS (**c**) with antibodies for fibrillin-1 and -2, podoplanin, and LYVE-1. Similar to the BPM, the BS of the patients with MH was more intensely stained for fibrillin-1 and -2 and for podoplanin than was the VC. In addition, the BPM and the BS of the patients with ERM were more intensely stained with antibodies for LYVE-1 than was the VC. The staining pattern of the BS closely resembled that of the BPM.

In Cases 5 to 8, the BS and BPM stained positive for fibrillin-1 and -2 (Figure 5b,c) whereas the VC stained negative (Figure 5a). The BS and BPM stained more intensely for podoplanin and LYVE-1 (Figure 5b,c) than did the VC (Figure 5a). Moreover, the staining patterns in BS and BPM were remarkably similar in appearance (Figure 5b,c).

## 3. Discussion

Although knowledge about the vitreous body has tremendously increased over the past several decades, the vitreous is still the most enigmatic tissue in the eye [18] due to its transparency and high water content, as mentioned above [1]. Hence, there has been a continuing controversy regarding the anatomical and physiological properties of the vitreous.

After a PVD is induced during vitrectomy, a thin membranous tissue remains adhered to the surface of the macular retina and it is usually associated with an oval or round defect in the posterior hyaloid membrane [19,20]. It has been generally accepted that this thin tissue is the posterior wall of the posterior precortical vitreous pocket, which Kishi et al. reported in 1990 using Trump’s fixed eyes and the fluorescein staining technique [21]. The posterior precortical vitreous pocket has been considered the same space as the BPM, and the posterior wall of the posterior precortical vitreous pocket has been regarded as the vitreous cortex [22,23]. However, in 1978, Worst described and assumed that, after complete PVD, the BPM might occasionally remain adhered to the macula, connecting to the canal extruded from the collapsed vitreous through the premacular oval defect in the posterior hyaloid membrane [8].

In 1989, Sebag also observed that the vitreous remained attached to the macula even in the presence of PVD, extruding through the hole in the posterior hyaloid membrane with fibers inserting into the macula [1,24]. Kakehashi et al. observed the vitreous with PVD associated with a premacular oval defect via the use of in vivo slit-lamp biomicroscopy and obtained findings similar to those described by Worst and Sebag [25]. Kakehashi et al. further reported that the Tyndall effect, an optical phenomenon, was mostly observed in the premacular vitreous without PVD, in which the BPM was supposed to be present, thus indicating that the formed vitreous gel occupied that space [26]. Consequently, we considered that the optically empty space that is observed by swept-source optical coherence tomography (SS-OCT) [27] is not actually empty.

In 2012, Polak, Ringens, and Worst observed the migration of intravitreally injected TA into the remaining thin tissue on the macula after artificial PVD [28] and postulated that this thin tissue might be the BPM, as the configuration of the migrated TA closely resembled that of the BPM and its connecting cisterns (i.e., the corona petaliformis of Worst), which was observed in vitro using the ink injection technique [5].

It has been generally recognized that idiopathic epiretinal membrane (ERM) is caused by fibrotic change of the posterior vitreous cortex remaining on the macula after complete PVD [19,22,29]. However, in 1975, Worst postulated that the BPM remained attached to the macula without connection to the collapsed vitreous after complete PVD and might contract, thus causing ERM [8]. In 1995, Okada et al. reported that ERM histologically consisted of collagen layer with or without a flattened cell layer [30]. Thus, we speculate that idiopathic ERM might be caused by fibrotic change of the complex of the posterior hyaloid membrane and the BPM (or its posterior wall) or the posterior hyaloid membrane alone that remains on the macula after PVD.

Vitreoschisis has been considered a lamellar split in the posterior vitreous cortex resulting from anomalous PVD [31,32]. Vitreoschisis and anomalous PVD were associated with various pathological conditions, such as a macular hole (MH), ERM, and proliferative diabetic retinopathy (PDR) [32]. Based on three-dimensional OCT (3D-OCT) findings, Sebag postulated that a multilamellar structure (i.e., of at least three layers) might be present in the posterior vitreous cortex, predisposing to vitreoschisis [18,32]. Sebag et al. [32] and Gupta et al. [33] also observed a lamellar structure of the posterior vitreous cortex in a monkey eye via staining with fluorescein-conjugated Agaricus bisporus agglutinin (ABA) lectin. However, the vitreous cortex above the fovea is extremely thin or absent [4,34,35,36]. Therefore, we assume that a multilamellar structure demonstrated by 3D-OCT imaging and by the fluorescein-labeled staining method might consist of the anterior and posterior walls of the BPM and the posterior hyaloid membrane and that vitreoshisis might be caused by fibrotic changes of these three membranous tissues associated with partial adhesion among them.

Kislitsyna et al. meanwhile reported that, after the removal of the anterior vitreous cortex in cadaver eyes, several membranes (i.e., four, on average; a multilamellar structure similar to the premacular vitreous) were detected covering the BS and with an ability to exfoliate (YouTube. Available at: https://www.evrs.eu/on-the-inner-side-of-lens-anatomic-and-topographic-features-of-anterior-vitreous-2/; minutes 2:03–5:00 (accessed on 31 May 2019)) [16,17]. Accordingly, the BS appears to share common anatomical features with the BPM.

The lymphatic vessels play vital physiological roles in tissue fluid balance, excretion of waste metabolites, immune defense, and transport of lipids [37]. One critical function of lymphatic vessels is to return excess interstitial fluid, which continually leaks out of the bloodstream through the permeable walls of blood capillaries, back to the blood circulation [38]. Only a few regions, including the bone marrow, epidermis, cartilage, and ocular tissues except for the ocular adnexa, are generally thought to be devoid of lymphatic vessels [39].

It has previously been reported that the central nervous system is an organ devoid of lymphatic vessels, thus raising long-standing questions about how excess water and waste metabolites are removed from the brain [40]. In 2012, Iliff et al. discovered a series of channels surrounding cerebral blood vessels that remove excess interstitial fluid and waste products such as amyloid-β, thus preventing the onset of Alzheimer disease [41]. This paravascular drainage system, managed by aquaporin-4 (AQP4) expressing astrocytes, was termed the glymphatic system [42]. Moreover, in 2015, Aspelund et al. [40] and Bucchieri et al. [43] discovered classic lymphatic vessels located in the dura mater of the brain meninges. In addition, the glymphatic system enables the brain to drain the interstitial fluid into the meningeal lymphatic vessels, draining to the deep cervical lymph nodes [44,45].

The retina, which is anatomically and developmentally known as an extension of the brain [46], has also been thought to be devoid of lymphatic vessels [47]. However, in 2015, Wostyn et al. and Denniston et al. reported the existence of a paravascular space (i.e., the glymphatic system) in the retina and the optic nerve, thus suggesting that, similar to Alzheimer’s disease, glymphatic pathway dysfunction might contribute to deficient amyloid-β clearance from the retina, ultimately causing glaucoma and age-related macular degeneration [48,49,50,51]. Similar to in the central nervous system, classic lymphatic vessels were recently found in the dura mater of the optic nerve [52].

Although the BPM has been regarded as an empty space, as mentioned above [27], our recent immunostaining study showed that the BPM might be a parenchymal tissue containing numerous cells expressing lymphatic endothelial cell markers podoplanin and LYVE-1; mast cell markers tryptase and chymase; and ER-TR7, a reticular fibroblast marker antibody [9]. Podoplanin and LYVE-1 are also implicated in uveal melanoma [53]. In this present study, the BPM was more intensely stained with antibodies for fibrillin-1 and -2 than was the VC (Figure 3, Figure 4 and Figure 5). Fibrillin-1 and -2 are the components of the lymph node conduits [54] that transport fluid containing immune cells and small molecules in the lymph nodes [55,56]. The conduit system has a collagen core that is surrounded by microfibrils containing fibrillin-1 and -2 and reticular fibroblasts expressing ER-TR7 [57,58]. It appeared that the entire BPM was stained, but since this specimen was whole-mounted, it is likely that only the surface of the BPM was stained. The lymph node sinuses are lined by a single layer of lymphatic endothelial cells expressing podoplanin and LYVE-1 [59,60]. Namely, the BPM specimens obtained during vitrectomy appeared to express several lymph node markers [9].

In our previous study, mast cells, which resided in the lymph nodes [61], were found in the BPM [9]. Worst showed that the BPM was expandable (YouTube. Available at: http://www.surgerytheater.com/video/9822/The-Vitreous-Body-Enigma-a-Paradigm-Shift-by-Prof-Dr-Jan-Worst; (accessed on 6 July 2012)), while the capsule of the lymph nodes has high distensibility [62,63]. Worst also showed that the cisternal system including the BPM collapsed after the posterior hyaloid membrane was removed from the surface, while the lymphatic vessels and the lymph nodes occasionally collapsed under non-physiological conditions [63,64,65]. Using a scanning electron microscope, Jongebloed et al. showed that a few bacteria were trapped in the BPM [66], which is a well-known phenomenon observed in the lymph nodes [67,68]. Consequently, our series of studies and a review of the literature seem to indicate the possibility that the BPM has similar properties to lymph nodes.

The findings in this present study showed that both the BS and the BPM are intensely stained with lymphatic markers, such as podoplanin, LYVE-1, and fibrillin-1 and -2 (Figure 5). The BS and the BPM closely resembled each other in regard to their staining patterns (Figure 3, Figure 4 and Figure 5) [9]. Furthermore, our intraoperative findings showed that the BS and the BPM had similar elastic properties. Kishi et al. also reported that the posterior wall of the posterior precortical vitreous pocket, which we consider the BPM, was elastic [35]. Judging from SS-OCT imaging, the BS and the BPM appear to be empty space, as previously described [35,36,69]; however, OCT images of the cortex of lymph nodes have been depicted as a low-scattering region compared with the surrounding connective tissue [70]. Therefore, it is possible that the BS could be composed of parenchymal tissue such as lymph nodes rather than empty space filled with fluid, as with the BPM [27].

Hemorrhage in the BS is occasionally observed after an episode of ocular trauma [11]. Moreover, studies have reported the occurrence of a sharply demarcated hemorrhage in the premacular region associated with several vitreoretinal diseases, such as PDR, Valsalva retinopathy, and Terson syndrome [71,72,73,74]. Premacular hemorrhage is thought to be located in the subhyaloid or sub-internal limiting membrane space [72,75]. However, based on the distribution of the blood, Jongebloed et al. postulated that premacular hemorrhage might occur within the BPM [76]. Meyer reported that the dome-shaped membrane located in front of a premacular hemorrhage was difficult to penetrate by yttrium aluminum garnet (YAG) laser [75], which we assume is the result of the elastic properties of the BPM capsule.

We previously reported that the removal of a hemorrhage adhering to the posterior lens capsule (presumably located within the BS) required resection with a vitreous cutter placed near the lens [77]. Moreover, Fine et al. and Shimada et al. reported that TA, which migrated (or was injected) into the BPM, could not be aspirated and that excision of the surrounding structure with a vitreous cutter was required to remove the TA [78,79]. Hence, it seems likely that the BS and the BPM might have sac-like structures with very similar features, thus exhibiting a mirror image across the vitreous body.

The periphery of the BS attaches to the posterior lens capsule via a thickened circular adhesion known as Wieger’s ligament [80,81]. The main component of Wieger’s ligament are oxytalan tissue fibers, and fibrillin-1 and -2 are known to be markers of those fibers [82]. However, Worst et al. reported the discovery of a perimacular vitreoretinal attachment ring (about 6 mm in diameter) that provides relatively strong adhesion between the BPM and the peripheral macula [83]. Shimada et al. reported that the BPM and Martegiani area were surrounded by a dense network of fibrils [84]. Our intraoperative findings showed that all three of these tissues had comparatively strong circular adhesions at the periphery with ligament-like structures, which were able to be separated from the retina or lens by aspiration with a vitreous cutter.

Using the ink-injection technique, Worst et al. showed that a septal structure, named the septum interpapillo-maculare, was clearly visible between the BPM and the Martegiani area [5,83]. Using SS-OCT, Itakura et al. reported a septal structure similar to the one observed by Worst [22]. When TA was injected into the BS, we observed that a septum-like structure divided the BS into a two-thirds temporal and a one-third nasal formation (Figure 2b, black arrowheads), presumably an extension of the septum interpapillo-maculare reported by Worst (YouTube. Available at: https://www.ophtec.com/company/blog/2018/10/849379-top-3-video-s-of-prof-dr-worst-on-youtube; 1. “The Vitreous Body Enigma. A Paradigm Shift” by Prof. Dr. Jan Worst; minutes 7:06–7:46 (accessed on 17 April 2012) [5].

Based on in vitro examination of the vitreous structure, Eisner theorized that the tracts of Eisner regulated the direction of intravitreal fluid transport [4,5]. Using the ink-injection technique, Worst observed two anteroposterior canals, i.e., the canalis ciliobursale (a canal between the retrociliary cisterns reported by Worst and the BPM) and the canalis optico-lenticulare (a wider funnel-shaped canal between the Martegiani area and the BS surrounding the Cloquet’s canal) [5,27] and postulated that they play an important role in the transport of fluid, nutrients, and waste products through the vitreous [5]. We theorize that the transport of excess fluid and nutrients (e.g., lipids) and the excretion of waste products are the specific roles of the lymphatic system [37].

It should be noted that the cisternal system appears to be irregular and expanded compared with the usual lymphatic vessels [5,83]. However, we assume that the configuration of the cisterns and bursa in the vitreous resemble that of the primitive lymph sacs [84] from which lymphatic vessels and lymph nodes develop during the embryonic period [85,86]. Eisner observed that the prefoveal *Lücke (lacuna or gap)* that emerged in the premacular vitreous after maturation of the fovea had begun, was always extended into the vitreous center as a canal, and even reached the anterior vitreous [34,83]. Kishi et al. reported that the posterior precortical vitreous pocket was restricted to a small premacular area in the vitreous of infants yet expanded anteriorly with age and occasionally occupied more than half of the vitreous [21]. Los, a colleague of Worst, postulated that an age-related increase in the volume of the cisternal system would be consistent with a differentiation process rather than matrix degeneration based on the observation of dye-injected vitreous of various ages [87]. We speculate that Eisner, Kishi, and Los observed nearly the same structure in the developing vitreous and that an increase in the volume of the structure with age could be an analogy of the development of the primitive lymph sacs. Based on the findings above, the cisternal system reported by Worst appears to resemble the lymphatic (or perilymphatic) system, both functionally and developmentally.

The lymphatic vessels begin with blind-ended initial lymphatics that are composed of a single layer of lymphatic endothelial cells [88]. The anchoring filaments extend radially from the outer surface of the endothelial cells and connect to the surrounding connective tissue, such as elastic fibers [89]. Increased interstitial fluid pressure and dynamic movement of the tissue stretch the anchoring filaments and create gaps between neighboring lymphatic endothelial cells, through which interstitial fluid flows unidirectionally into the lymphatic vessels [90]. As we described above, podoplanin, LYVE-1, and fibrillin-1 and -2, which were all detected in the BS and BPM specimens, are components of lymph nodes. However, podoplanin and LYVE-1 are expressed in the lymphatic endothelial cells in the initial lymphatics [91] and the anchoring filaments mainly consist of fibrillin-1 and -2 [92,93]. Hence, it is possible that the initial lymphatic-like structure might exist in the BS and the BPM.

Worst et al. reported that the BPM surface is feathery or is surrounded by fine radiating lines [5]. When TA was injected into the posterior part of the vitreous during vitrectomy, the TA particles adhered exclusively to the anterior surfaces of the BPM and Martegiani area, presumably due to their feathery structure (Figure 1b) [5]. Furthermore, the surface appearance of the BPM described by Worst et al. [5] seems to resemble that of the initial lymphatics associated with the anchoring filaments [94]. Assuming that the surfaces of the BS and the BPM consist of an initial lymphatic-like structure, excess vitreous fluid might enter these tissues via increased intraocular pressure (IOP) and movement of the vitreous.

If our assumption regarding the function of the BS and the BPM is correct, the fluid entering these tissues would need to drain out of the eye. Until the 1960s, the theory of ocular lymph drainage had generally been discarded [95]. However, since then, several studies have shown that intravitreally injected materials, including India ink, radioactive tracers, and liposomes, drained into the cervical lymph nodes [95,96,97,98]. Those findings indicate that a communication between the vitreous cavity and the general lymphatic system might exist.

It is generally accepted that the aqueous humor produced in the ciliary body exits the eye either through the trabecular meshwork into the Schlemm’s canal (i.e., the conventional pathway) or through the ciliary muscle and other downstream tissues (i.e., the uveoscleral pathway) [99]. However, in 2009, the findings of Yücel et al. using lymphatic markers, podoplanin, and LYVE-1 proved the presence of lymphatic vessels in the ciliary body, thus suggesting that they are a third pathway for the drainage of aqueous humor [100].

We assume that the fluid entering the BPM might flow into the retrociliary cisterns through the canalis ciliobursale reported by Worst. Since the retrociliary cisterns are adjacent to the ciliary body [86], the fluid that enters the retrociliary cisterns might drain out into the lymphatic vessels in the ciliary body, draining into the superficial cervical lymph nodes [100,101].

The vitreous cavity is generally considered a closed system, with no direct interaction with the retrobulbar subarachnoid space [102]. However, numerous studies have investigated the drainage of the vitreous humor into the optic nerve [103]. In 1879, Kuhnt was the first to suggest that the papillary edema in elevated intracranial pressure was due to the stasis of lymphatic flow into the optic nerve sheath [104]. In 1884, Ulrich reported that the vitreous humor flowed into the optic nerve and then into the orbit of rabbits, and this findings was confirmed in other rabbit-model studies [105]. In 1979, Algvere reported that plastic microspheres injected into the central vitreous of rabbits moved toward the optic disc, where they subsequently converged around the central retinal artery [106].

The foveal retina is considered devoid of a paravascular channel, a component of the glymphatic system [107], due to its avascularity. However, a drainage pathway other than a paravascular space must be present to remove excess fluid from the fovea, as previously described. In 2018, Behar-Cohen et al. found that glial fibrillary acidic protein (GFAP)-positive cells that coexpress AQP4 are abundantly present at the roof of the human fovea [108]. In 2019, Ikeda et al. reported that the inner layer of the monkey fovea was mostly composed of GFAP-intensely positive astrocytes [109]. In 2020, Delaunay et al. reported that, in humans, connexin-43 and GFAP double-positive astrocytic glia cells cover the foveal pit [110]. Since AQP4-positive astrocytes contribute to the glymphatic system [108,111], a glymphatic clearance pathway associated with astrocytes could exist at the surface of the fovea.

Frisina et al. reported frequent observation of persistent or new cystoid macular edema (CME) following vitrectomy for ERM [112]. Worst postulated that the pathology of CME might be due to detachment of the BPM from the macula [8]. These findings indicate the possibility that the BPM might participate in the glymphatic clearance pathway involving astrocytes of the fovea and that excess interstitial fluid in the fovea might flow into the cisternal system via the BPM.

It should be noted that one limitation in this present study is that it was technically difficult to collect the BS and BPM specimens in an intact form during vitrectomy, thus explaining why we collected the specimens by cutting them into small pieces with a vitreous cutter. Hence, the BPM specimens that were obtained may have contained other tissues, including the Martegiani area, the septum interpapillo-maculare, and the posterior hyaloid membrane. We speculate that this is the reason why the staining patterns of the BPM were extremely different among the specimens, as shown in Figure 3. We consider that the BS as well as the BPM might be parenchymal tissues and that the histological findings of the specimens might reflect their inner structures. However, it is quite possible that these tissues might consist of hollow structures and that the specimens might be their walls. Therefore, it will be necessary to conduct further immunohistological studies using human cadaver eyes to determine the fine structures of the BS and the BPM.

The retrolental bursa described by Kislitsyna (i.e., the BS) and the premacular bursa described by Worst are adjacent to the posterior part of the lens and the macula, respectively, where pathological changes are frequently observed. Consequently, these tissues would be the most important parts in the vitreous, both physiologically and pathologically. Further investigation on the precise anatomy and physiological function of these tissues may provide clues to elucidate the pathogenesis mechanism of various ocular diseases, including cataracts, macular diseases, and glaucoma. It should be noted that it would be meaningful to study the relationship between such lymphoid tissues and the eye.

## 4. Materials and Methods

### 4.1. Collection of the BS, BPM, and VC Specimens

During vitrectomy, BPM and VC specimens were selectively collected from 6 eyes of 6 patients with MH (1 male and 5 females, age range: 62–79 years) and 2 eyes of 2 patients with ERM (1 male and 1 female, age 63 and 77 years, respectively) in which the BPM was clearly identified. All of those patients underwent both vitrectomy and cataract surgery at the same time. The vitreous specimens were collected in the same manner as previously described by Sato et al. [9]. Using a 25-gauge (G) transconjunctival sutureless vitrectomy system, the VC was collected with a vitreous cutter before the start of perfusion. To collect the BPM specimens, the vitreous was extensively resected, and TA was applied to visualize the BPM and the Martegiani area. Holes were then created in the anterior and posterior walls of the BPM with a diamond scraper. From these holes, vitreous fluid spontaneously flowed into the space between the BPM and the retina, resulting in the BPM being easily separated from the retinal surface by aspirating the edge of the hole in the anterior wall of the BPM with a vitreous cutter. Since the Martegiani area was connected to the BPM near the optic disc with a septal structure, both tissues were removed from the retina en bloc. Subsequently, the perfusate in the tube connected to the vitreous cutter was completely removed by injecting air into the connector in the reverse direction. The cutter was then reinserted into the vitreous cavity, and the BPM floating above the macula was selectively collected by reducing the rotation speed to 500/min.

From 4 eyes of 4 patients with MH (1 male and 3 females, age range: 64–75 years), the BS specimens were collected in addition to the BPM and VC specimens following the previously described method. To collect the BS specimens, TA was injected into it from behind under direct microscopy to visualize the interface between the BS and the surrounding vitreous. The posterior wall of the BS was then aspirated with a vitreous cutter and detached from the lens. After the perfusate in the tube was completely removed, as with the case of the BPM, the cutter was reinserted into the vitreous cavity to selectively collect BS specimens floating behind the lens. Informed written consent was obtained from all patients for involvement in the study.

### 4.2. Immunostaining of the BPM and VC with Antibodies for Fibrillin-1 and -2

Immunostaining with antibodies for fibrillin-1 (rabbit polyclonal, 1:300; Bioss Antibodies, Inc., Woburn, MA) and fibrillin-2 (rabbit polyclonal, 1:300; Bioss Antibodies) was performed in 3 eyes of 3 patients with MH (Cases 1–3) and in 1 eye of 1 patient with ERM (Case 4). After collection, the BPM and VC specimens were fixed with 4% paraformaldehyde, rinsed with phosphate buffer saline (PBS, pH 7.4), and blocked with 5% normal goat serum. Immunostaining was then performed by the avidin–biotin complex method with each target antibody. Next, the BPM and VC specimens were incubated with antibodies for fibrillin-1 and -2 at 4 °C for 2 days, rinsed with PBS, and incubated with biotinylated anti-rabbit immunoglobulin G (1:1000; Vector Laboratories, Inc., Burlingame, CA) at room temperature for 2 h. After a rinse with PBS, the specimens were incubated with alkaline phosphatase-labeled avidin–biotin complex (Vector Laboratories) at room temperature for 2 h and treated with an ImmPACT™ Vector^®^ Red alkaline phosphatase substrate (Vector Laboratories). After dehydration, the specimens were enclosed with Entellan^®^ new (Merck, KGaA, Darmstadt, Germany) and observed under a BZ-x700 microscope (Keyence Corporation, Osaka, Japan).

### 4.3. Immunostaining of the BS, BPM, and VC Specimens with Antibodies for Fibrillin-1 and -2, Podoplanin, and LYVE-1

BS, BPM, and VC specimens were selectively collected from 4 eyes of 4 patients with MH and ERM (Cases 5–8), and each specimen was immunostained with antibodies for fibrillin-1 and -2, podoplanin (rabbit polyclonal, 1:500; Abcam Inc., Cambridge, UK), and LYVE-1 (rabbit polyclonal, 1:500; Abcam). The BS, BPM, and VC specimens were stained in the manner described above.

## Data Availability

The data presented in this study are available on request from the corresponding author.

## Abbreviations

BPM	bursa premacularis
PVD	posterior vitreous detachment
LYVE-1	lymphatic vessel endothelial hyaluronan receptor 1
BS	Berger’s space
AHM	anterior hyaloid membrane
TA	triamcinolone acetonide
VC	vitreous core
SS-OCT	swept-source optical coherence tomography
ERM	epiretinal membrane
MH	macular hole
PDR	proliferative diabetic retinopathy
3D-OCT	three-dimensional optical coherence tomography
ABA	Agaricus bisporus agglutinin
AQP4	aquaporin-4
SD-OCT	spectral domain optical coherence tomography
YAG	yttrium aluminum garnet
IOP	intraocular pressure
RPE	retinal pigment epithelial
GFAP	glial fibrillary acidic protein
CME	cystoid macular edema
PBS	phosphate buffered saline
